# S-shaped Kidney: A Rare Occurrence of Supernumerary Kidney with Fusion-malrotation

**DOI:** 10.5334/jbsr.1666

**Published:** 2018-11-23

**Authors:** Wouter Mebis, Benjamin Peters, Thijs van der Zijden

**Affiliations:** 1 Antwerp University Hospital, BE

**Keywords:** S-shaped kidney, supernumerary, fusion, malrotation, computed tomography

## Case

**Figure 1:**
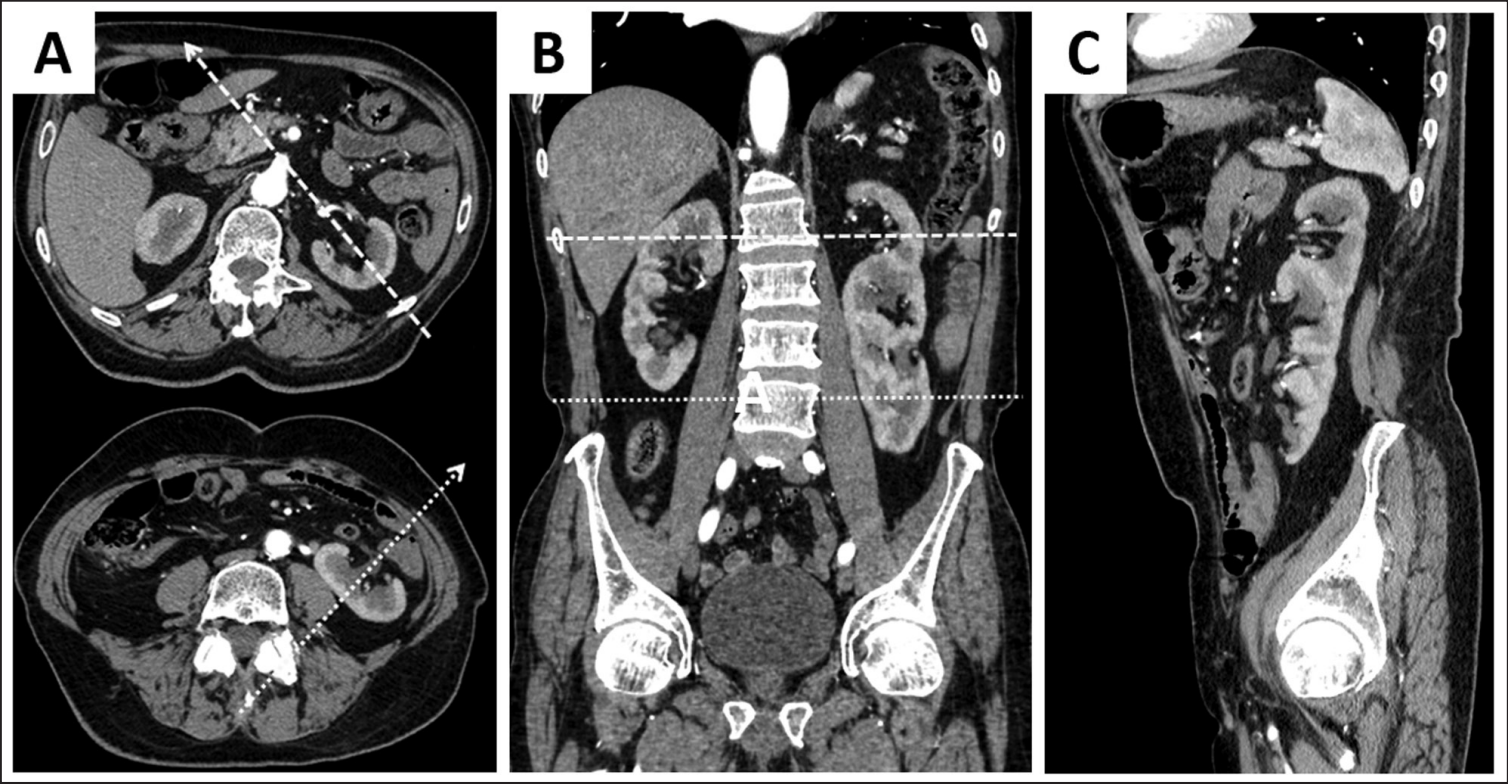
Contrast-enhanced CT with axial **(A)**, coronal **(B)** en sagittal **(C)** reformations demonstrating a large, S-shaped kidney in the left renal fossa and a normal kidney on the right.

**Figure 2:**
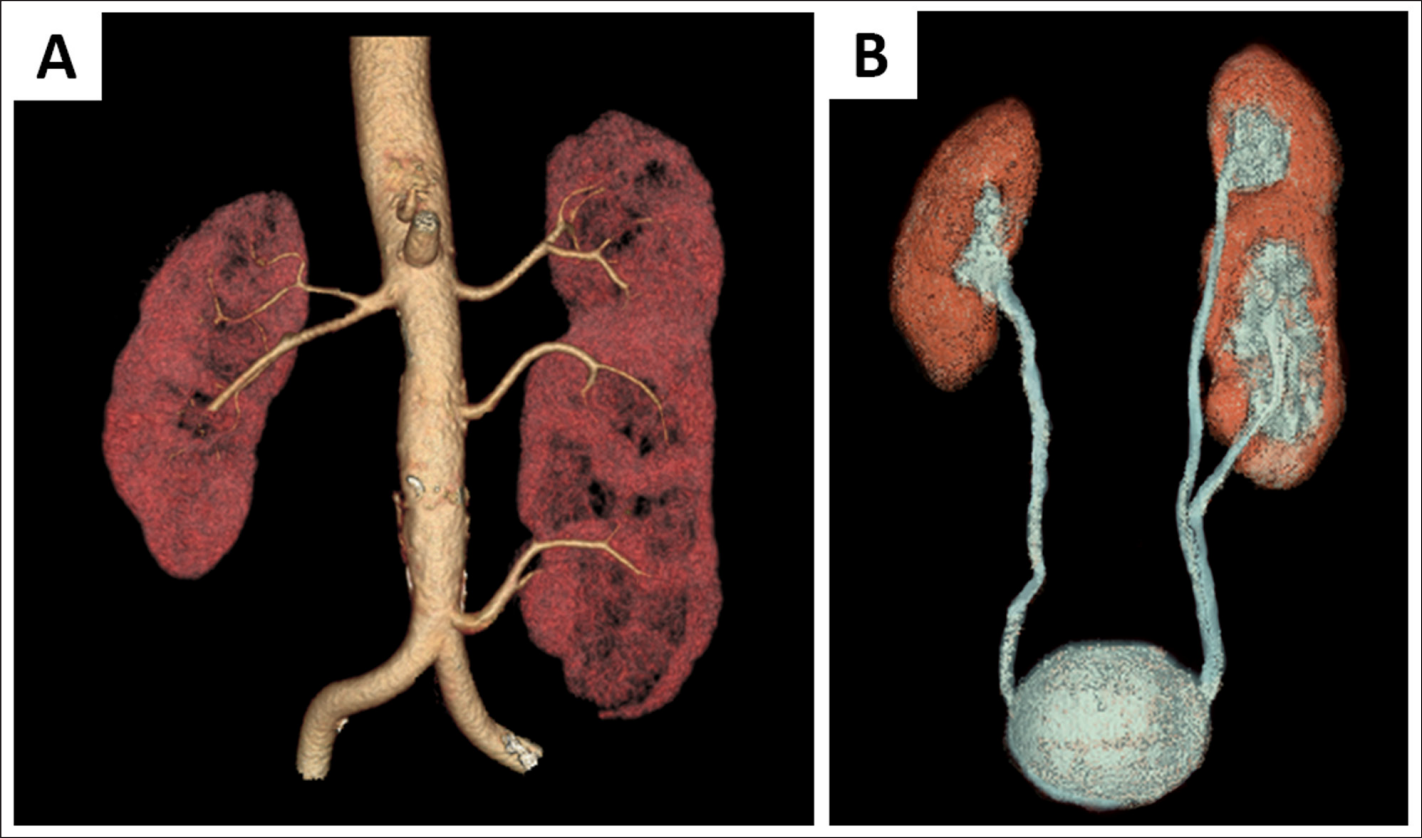
3D volume rendering of the renal arteries **(A)** and the excretory system **(B)**. The left kidney is supplied by 3 renal arteries **(A)** and has two separate ureters **(B)**.

A 50-year-old man was admitted to the emergency department with abdominal pain. Blood testing showed signs of inflammation. Renal function was normal. Computed tomopgraphy (CT) of the abdomen was performed after intravenous contrast injection (Figure 1 A–C). No clear explanation for the abdominal pain was found. However, there was a small supernumerary kidney fused on top of a normal-sized left kidney. The upper kidney had a normal, anteromedially oriented pelvis but the lower kidney had an anterolaterally oriented pelvis (dotted arrows, Figure 1A) rendering an S-shaped kidney. There were two separate but closely related ureters, as shown on the three-dimensional volume rendering (3D VR) of the excretory system (Figure 2A). Three left renal arteries could be seen: one at the normal level supplying the upper part and two at a lower level supplying the lower kidney, best seen on the 3D VR of the renal arteries (Figure 2B). Multiple interconnected renal veins were seen forming three common trunks, draining into the inferior vena cava and left common iliac vein (not shown). The right kidney had a normal shape, single vascular supply and ureter.

## Comment

A supernumerary kidney is a rare congenital anomaly with less than 100 cases documented. It can be separate from the normal kidneys or partially fused. Supernumerary kidneys are generally smaller and more frequently seen on the left side [1]. Although the duplex kidney is more common, a supernumerary kidney can be seen as a separate organ with its own vascular supply and collecting system as well as a distinct capsule. In contrast, a duplex kidney is contained within a single renal capsule and has the same (single) vascular supply as the contralateral kidney. The total number of calices in a supernumerary kidney and the ipsilateral kidney is higher than in a duplex kidney and exceeds that of the contralateral kidney [1].

The possible cause could be found in the embryonal development. Normally, a single metanephric blastema is formed within the nephrogenic cord and a single ureteral bud grows towards it, forming a single kidney. When one ureteral bud bifurcates or two separate ureteral buds penetrate a single metanephric blastema it results in a duplex kidney. However, when two metanephric blastemas are formed and they are penetrated separately by a bifurcated or double ureteral bud, a supernumerary kidney is formed [1].

In our case there was an additional rotation anomaly with hyper rotation of the lower kidney. This is thought to be caused by a late or abnormal insertion of the ureteral bud into the metanephric blastema, impairing normal rotation [1].

Supernumerary kidneys (and other congenital renal anomalies) can present with hydronephrosis, pyelonephritis, urolithiasis and other pathologies in over 50% of cases. Associated genital, gastrointestinal, central nervous system and musculoskeletal anomalies have also been described [1].

Diagnosis can be made with ultrasound, computed tomography and magnetic resonance imaging [1].

No treatment is required unless the patient is symptomatic [1].
